# Defects of mtDNA Replication Impaired Mitochondrial Biogenesis During *Trypanosoma cruzi* Infection in Human Cardiomyocytes and Chagasic Patients: The Role of Nrf1/2 and Antioxidant Response

**DOI:** 10.1161/JAHA.112.003855

**Published:** 2012-12-19

**Authors:** Xianxiu Wan, Shivali Gupta, Maria P. Zago, Mercy M. Davidson, Pierre Dousset, Alejandro Amoroso, Nisha Jain Garg

**Affiliations:** Department of Microbiology and Immunology, University of Texas Medical Branch, Galveston, TX (X.W., S.G., N.J.G.); Department of Pathology, University of Texas Medical Branch, Galveston, TX (N.J.G.); Faculty of the Institute for Human Infections and Immunity and the Center for Tropical Diseases, University of Texas Medical Branch, Galveston, TX (N.J.G.); Instituto de Patologia Experimental-Consejo Nacional de Investigaciones Científicas y Técnicas (IPE-CONICET), Facultad de Ciencias de la Salud, Universidad Nacional de Salta, Salta, Argentina (M.P.Z.); Department of Radiation Oncology, Columbia University, New York, NY (M.M.D.); Servicio de Cirugia Cardiovascular, Hospital San Bernardo, Salta, Argentina (P.D., A.A.)

**Keywords:** Chagas disease, mitochondrial biogenesis, mtDNA replication, NRF2, oxidative stress, PGC-1α, *Trypanosoma cruzi*

## Abstract

**Background:**

Mitochondrial dysfunction is a key determinant in chagasic cardiomyopathy development in mice; however, its relevance in human Chagas disease is not known. We determined if defects in mitochondrial biogenesis and dysregulation of peroxisome proliferator-activated receptor gamma (PPARγ) coactivator-1 (PGC-1)–regulated transcriptional pathways constitute a mechanism or mechanisms underlying mitochondrial oxidative-phosphorylation (OXPHOS) deficiency in human Chagas disease.

**Methods and Results:**

We utilized human cardiomyocytes and left-ventricular tissue from chagasic and other cardiomyopathy patients and healthy donors (n>6/group). We noted no change in citrate synthase activity, yet mRNA and/or protein levels of subunits of the respiratory complexes were significantly decreased in *Trypanosoma cruzi*–infected cardiomyocytes (0 to 24 hours) and chagasic hearts. We observed increased mRNA and decreased nuclear localization of PGC-1-coactivated transcription factors, yet the expression of genes for PPARγ-regulated fatty acid oxidation and nuclear respiratory factor (NRF1/2)–regulated mtDNA replication and transcription machinery was enhanced in infected cardiomyocytes and chagasic hearts. The D-loop formation was normal or higher, but mtDNA replication and mtDNA content were decreased by 83% and 40% to 65%, respectively. Subsequently, we noted that reactive oxygen species (ROS), oxidative stress, and mtDNA oxidation were significantly increased, yet NRF1/2-regulated antioxidant gene expression remained compromised in infected cardiomyocytes and chagasic hearts.

**Conclusions:**

The replication of mtDNA was severely compromised, resulting in a significant loss of mtDNA and expression of OXPHOS genes in *T cruzi–*infected cardiomyocytes and chagasic hearts. Our data suggest increased ROS generation and selective functional incapacity of NRF2-mediated antioxidant gene expression played a role in the defects in mtDNA replication and unfitness of mtDNA for replication and gene expression in Chagas disease.

## Introduction

Chagas disease is a major health concern in the South American continent and an emerging infectious disease in the United States. Clinical symptoms progress from hypertrophic remodeling (wall thickening) to dilated cardiomyopathy that ultimately results in cardiac arrest and death.^1^ No effective therapies are available for the treatment of the migrated workforce or the 20 million infected individuals living in the endemic countries.

Experimental studies suggest mitochondrial function is impaired in chagasic hearts. Attachment and invasion by *Trypanosoma cruzi* alters plasma membrane and induces intracellular Ca^2+^ flux.^2^ It is suggested that these events contributed to the mitochondrial permeability transition pore (MPTP) opening, leading to a decline in respiratory chain activity in cardiomyocytes^3^ and heart tissue of *T cruzi–*infected mice and rats.^4,5^ Consequently, an increase in electron leakage to O_2_ and O_2_^•−^ formation^6^ associated with a decline in ATP production^7^ was observed in cardiomyocytes and heart tissue of infected rodents. Furthermore, mtROS signaled the NFκB pathway of cytokine gene expression in infected cardiomyocytes.^8,9^ These studies suggested that mitochondrial metabolic abnormalities contribute to energy deficiency, oxidative stress, and possibly an inflammatory state of the heart and may not just be secondary events related to the pathology of cardiac remodeling and heart failure in Chagas disease. The molecular mechanisms responsible for these pathological conditions are not understood, however, making it difficult to develop therapies for control of mitochondrial dysfunction and the associated adverse effects in chagasic (and other) heart disease.

Recent studies have suggested a central role of peroxisome proliferator-activated receptor gamma (PPARγ) coactivator-1α (PGC-1α) in mitochondrial biogenesis and function (reviewed in reference ^10^). PGC-1α has been shown to bind PPARs that are key regulators of genes involved in fatty acid oxidation.^11^ Nuclear respiratory factors (NRF1/2) are also coactivated by PGC-1α, driving the expression of genes involved in oxidative phosphorylation (OXPHOS), transcription, and replication of the mitochondrial genome and antioxidant gene expression.^12^ Estrogen-related receptors (ERRs) serve as an amplifier for PGC-1α activation of PPARs and NRFs. Several studies indicate that PGC-1 plays a critical role in normal cardiac development and function in animal models (reviewed in reference ^10^). However, the respective role of PGC-1-driven pathways has not been characterized in cardiomyopathy of chagasic or other etiologies in humans.

In this study, we chose to investigate whether dysregulation of PGC-1α transcriptional cascade(s) is the index event triggering mitochondrial abnormalities in *T cruzi–*infected cardiomyocytes and human chagasic hearts. We aimed to determine if *T cruzi–*infected cardiomyocytes and human hearts exhibit defective mitochondrial biogenesis and whether PGC-1α-mediated transcriptional regulation of genes associated with mitochondrial replication/transcription and OXPHOS and antioxidant status were aborted. Our results showed that mtDNA content and mtDNA-encoded transcription of genes of the OXPHOS pathway were significantly decreased in *T cruzi–*infected cardiomyocytes and chagasic hearts. We found that mtDNA was oxidized and unfit to carry out mtDNA replication in infected cardiomyocytes, and these defects were not related to PGC-1α, but were associated with nonresponsiveness of the NRF1/2 pathway of antioxidant gene expression in Chagas disease.

## Materials and Methods

### Antibodies and Reagents

All gene abbreviations are defined in Table 1. Polyclonal antibodies against PGC-1α (ab72230), PPARγ (ab19481), ERRα (ab76228), ERRγ (ab82319), and monoclonal anti-SDHA antibody (ab14715) were purchased from ABCAM (Cambridge, UK). Polyclonal antibodies against ND1 (sc-20493), NRF2 (sc-722), CYTB (H-300), TFB2M (sc-160858), SOD1 (FL-154), and SOD2 (FL-222) were purchased from Santa Cruz Biotech (Santa Cruz, CA). Antibodies against 4-HNE (ab5605), 3-NT (MAB5404), and 8-OHdG (ab3560) were from Millipore (Billerica, MA), and anti-β-actin antibody (A5441) was purchased from Sigma-Aldrich (St. Louis, MO). All chemicals were of molecular grade and were purchased from Sigma-Aldrich.

**Table 1.** Oligonucleotides Used in This Study

Gene Name	Protein Name	Genbank Accession #	Oligonucleotide	Oligonucleotide Sequence 5′–3′	Amplicon Size (bp)
*PGC-1α*	PPARγ coactivator-1α	NM_013261.3	PGC1α F	GTCACCACCCAAATCCTTAT	131
			PGC1α R	ATCTACTGCCTGGAGACCTT	
				
*PRC*	PGC-1-related coactivator	NM_015062.3	PRC F	GCAACGCCAAGCAGAAACAGAAGA	115

			PRC R	TGGTGGGATGACAAGACAAGGGAT	
				
*ERRα*	Estrogen-related receptor α	NM_004451.3	ERRα F	GGCAAAGTGCTGGCCCATTTCTAT	80

			ERRα R	TCGAGCATCTCCAAGAACAGCTTG	
				
*PPARγ*	Peroxisome proliferator-activated receptor γ	NM_138711.3	PPARγ F	GGCTTCATGACAAGGGAGTTTC	74

			PPARγ R	AACTCAAACTTGGGCTCCATAAAG	
				
*NRF1*	Nuclear respiratory factor 1	NM_001040110.1	NRF1 F	GGCACTGTCTCACTTATCCAGGTT	115

			NRF1 R	CAGCCACGGCAGAATAATTCA	
				
*NRF2*	Nuclear respiratory factor 2	NM_001197297.1	NRF2 F	CAGCCTGAACTGGTTGCACAGAAA	190

			NRF2 R	TCAACTCCGCTGCACTGTATCCAA	
				
*NRF2β*	Nuclear respiratory factor 2β	NM_005254.5	NRF2B F	TGAAACGGGTGTATCTGCTG	180

			NRF2B R	GAACTAACTACTTGCTGAATGGC	
				
*POLRMT*	RNA polymerase, mt	NM_005035.3	POLRMT F	GACATGTACAACGCCGTGATGCTT	91

			POLRMT R	AGCCGGCATCCTTCACCATGAATA	
				
*TFB1M*	Transcription factor B1, mt	NM_016020.3	TFB1M F	GGACACTCGATTTATTCCTGGATT	78

			TFB1M R	ACATCTCCATGAACAATTCTCAGTTT	
				
*TFB2M*	Transcription factor B2, mt	NM_022366.2	TFB2M F	TCTGGCAATTAGCTTGTGAGATTAA	101

			TFB2M R	CCTACGCTTTGGGTTTTCCA	
				
*TFAM*	Transcription factor A, mt	NM_003201.1	TFAM F	AATGGATAGGCACAGGAAACC	136

			TFAM R	CAAGTATTATGCTGGCAGAAGTC	
				
*MCAD*	Medium-chain acyl CoA dehydrogenase	NM_001127328	MCAD F	ATGGGCCAGCGATGTTCAGATACT	101

			MCAD R	GCAACTTTGAAACCAGCTCCGTCA	
				
*CKMT2*	Creatine kinase, mt 2	NM_001151.3	CKMT2 F	AAGAACGAGGCTGGGAGTTCATGT	132

			CKMT2 R	AGCTTTGGGATCCTAACGTGGACA	
				
*ND1*	NADH dehydrogenase complex subunit 1	NM_004541.3	ND1 F	CTGGCTACTGCGTACATCCA	142

			ND1 R	TCTCCAAACCCTTTGACACA	
				
*ND4*	NADH dehydrogenase complex subunit 4	NM_002495.2	ND4 F	AGGACTTCCACATGGAGATTGGCA	162

			ND4 R	AGACTGCATGTTATTGCGAGCAGG	
				
*SDHB*	Succinate dehydrogenase subunit B	NM_003000.2	SDHB F	CCACAGCTCCCCGTATCAAG	170

			SDHB R	TCGGAAGGTCAAAGTAGAGTCAA	
				
*CytB*	Cytochrome B	NC_012920.1	CytB F	AGTCCCACCCTCACACGATTCTTT	185

			CytB R	AGTAAGCCGAGGGCGTCTTTGATT	
				
*CytC*	Cytochrome C	NM_018947.5	CytC F	TGGGCCAAATCTCCATGGTCTCTT	86

			CytC R	TGCCTTTGTTCTTATTGGCGGCTG	
				
*UQCRC2*	Ubiquinol-cytoc C reductase protein II	NM_003366.2	UQCRC2 F	TTCAGCAATTTAGGAACCACCC	119

			UQCRC2 R	GTCACACTTAATTTGCCACCAAC	
				
*COI*	Cytochrome oxidase complex subunit I	NC_012920.1	COI F	ACCCTAGACCAAACCTACGCCAAA	90

			COI R	TAGGCCGAGAAAGTGTTGTGGGAA	
				
*COXIV*	Cytochrome oxidase complex subunit IV	NM_001861.3	COXIV F	TTTAGCCTAGTTGGCAAGCGA	105

			COXIV R	CCGATCCATATAAGCTGGGAGC	
				
*16S*	16S rRNA	NC_011137.1	16S F	CGCATAAGCCTGCGTCAGATAAAA	103

			16S R	TGTGTTGGGTTGACAGTGAGGGTA	
				
*ATP5A*	ATP synthase complex subunit 5A	NM_001001937.1	ATP5A F	TACATGGGCTGAGGAATGTTCA	179

			ATP5A R	ACCAACTGGAACGTCCACAAT	
				
*CAT*	Catalase	NM_001752.3	CAT F	TAAGACTGACCAGGGCATC	201

			CAT R	CAAACCTTGGTGAGATCGAA	
				
*GPx-1*	Glutathione peroxidase-1	NT_022517.18	GPx-1 F	AGCCCAACTTCATGCTCTTC	401

			GPx-1 R	CAGGTGTTCCTCCCTCGTAG	
				
*HO-1*	Heme oxygenase 1	NM_002133.2	HO-1 F	CAGGCAGAGAATGCTGAGTTC	271

			HO-1 R	GCTTCACATAGCGCTGCA	
				
*MnSOD*	Mn^2+^ superoxide dismutase	NM_001024466.1	MnSOD F	ACAGGCCTTATTCCACTGCT	168

			MnSOD R	CAGCATAACGATCGTGGTTT	
				
*BAX*	Bcl-2-associated X	NM_138764.4	BAX F	CATGTTTTCTGACGGCAACTTC	107

			BAX R	AGGGCCTTGAGCACCAGTTT	
				
*BCL2*	B-cell lymphoma 2	NM_000657.2	BCL2 F	GGTGGTGGAGGAGCTCTTCA	92

			BCL2 R	TGACGCTCTCCACACACATGA	
				
*GAPDH*	Glyceraldehyde 3-P dehydrogenase	NM_002046.3	GAPDH F	CCACTCCTCCACCTTTGAC	102

			GAPDH R	ACCCTGTTGCTGTAGCCA	
				
*SSBP1*	Single-stranded DNA-binding protein	NM_003143.1	SSBP1 F	TGCTCGGGTTAGATCGTCAGGAAA	175

			SSBP1 R	GCCCAAGTAAGTGCACACGATTCA	
				
*POLG*	Polymerase gamma, mt	NM_001126131.1	POLG F	TGTCAACCAGAACTGGGAGCGTTA	95

			POLG R	TGGCCAGATCCATCAACGACTTCT	
				
*PEO1*	Progressive external ophthalmoplegia 1	NM_001163812.1	PEO1 F	ATTGTAGAAGGACGTGGACGCGAA	123

			PEO1 R	TGCAGAGCTCACTCTAGGTGCATT	
				
*TOP1mt*	Topoisomerase DNA I, mt	NM_052963.1	TOP1 F	TTATCCTACAACCGAGCCAACCGA	115

			TOP1 R	TCTTTGCCTGGATCTTCGTCTGGA	
				
7S DNA	7S DNA, mt	NC_012920.1	7S DNA F	ATCAACTGCAACTCCAAAGCCACC	184

			7S DNA R	TGATTTCACGGAGGATGGTGGTCA	
				
18S rRNA	18S ribosomal RNA	NT_167214.1	18S rRNA F	GTAACCCGTTGAACCCCATT	147

			18S rRNA R	CCATCCAATCGGTAGTAGCG	

### Parasites and Infection

*Trypanosoma cruzi* trypomastigotes (SylvioX10/4) were propagated in C2C12 cells in RPMI 1640 medium with 5% FBS. AC16 (human ventricular cardiomyocyte) cells seeded in 6-well plates (5×10^4^/well) or T75 flasks (3×10^6^/flask, 70% confluence) were infected with *T cruzi* trypomastigotes (cell:parasite ratio 1:3) and incubated at 37°C, 5% CO_2_ for 0, 3, 6, 12, and 24 hours.

### Human Samples

All procedures for human sample collection were approved by the institutional review boards at the University of Texas Medical Branch (ID: 04-257) and the Universidad Nacional de Salta (UNSa), Argentina. Seropositivity for *T cruzi*–specific antibodies was confirmed by 2 serology tests.^7^ Clinical data included medical history, physical examination, subjective complaint of frequency and severity of exertional dyspnea, electrocardiography (at rest and with exercise) to reveal cardiac rhythm and conduction abnormalities, transthoracic echocardiogram to analyze left ventricular (LV) contractile function, and chest x-ray to assess cardiomegaly (cardiothoracic ratio >0.5). Cardiac biopsies were obtained from cardiomyopathy patients exhibiting systolic dysfunction (EF ≤40% to 55%) and/or left ventricular end diastolic diameter ≥57 mm, requiring correctional surgical intervention for clinical purposes at the San Bernardo Hospital, Salta. Normal cardiac biopsies were obtained from the National Disease Research Interchange tissue bank (Philadelphia, PA).

Subjects with comorbid diseases, for example, cancer, autoimmune disorders, neurodegenerative diseases, hepatic, renal chronic disease, chronic obstructive pulmonary disease, other parasitic infection (*Leishmania*), alcoholism, drug abuse history, or hematological disease and those individuals who were HIV positive were not included in the study. The exclusion criteria were employed to rule out the effects of comorbid diseases on the results obtained from infection by *T cruzi* and Chagas disease.

### Homogenates and Fractionation

Cardiomyocytes (6×10^6^ cells/mL, normal and infected) were incubated on ice for 30 minutes in lysis buffer constituting 50 mmol/L Tris (pH 7.5), 150 mmol/L NaCl, 1 mmol/L EDTA, 1 mmol/L EGTA, 1% Nonidet P-40, 2.5 mmol/L KH_2_PO_4_, and 1 mmol/L each of glycerophosphate, NaF, and Na_3_VO_4_. Cell lysates were centrifuged at 3000*g* at 4°C for 15 minutes and the resultant supernatants stored at −20°C**.**

For the preparation of nuclear and cytosolic fractions, cells (6×10^6^/mL) were incubated on ice for 30 minutes in buffer A (10 mmol/L HEPES [pH 7.9], 10 mmol/L NaCl, 0.1 mmol/L EDTA, 0.1 mmol/L EGTA, 1 mmol/L DTT, 1 mmol/L PMSF) containing 0.625% NP-40. Cell lysates were centrifuged at 4°C at 10 000*g* for 1 minute and supernatants stored as a cytosolic fraction. Pellets were washed with buffer B (1.7 mol/L sucrose, 10 mmol/L HEPES [pH 7.9], 10 mmol/L NaCl, 0.1 mmol/L EDTA, 0.1 mmol/L EGTA, 1 mmol/L DTT, and 1 mmol/L PMSF), resuspended in buffer C (20 mmol/L HEPES [pH 7.9], 0.4 mol/L NaCl, 1 mmol/L EDTA, 1 mmol/L EGTA, 1 mmol/L DTT, and 1 mmol/L PMSF), and centrifuged at 4°C at 13 000*g* for 5 minutes. The resultant supernatants were stored at −20°C as nuclear extracts.

### Real-Time RT-PCR

Total RNA from cardiomyocytes (6×10^6^/sample) was extracted using an RNeasy Mini Kit (Qiagen), and cDNA was synthesized using an iScript cDNA Synthesis Kit (Bio-Rad). First-strand cDNA was used as a template in a real-time PCR on an iCycler thermal cycler with SYBR Green Supermix (Bio-Rad) and gene-specific oligonucleotides, listed in Table 1. The threshold cycle (*C*_*t*_) values for each target mRNA were normalized to *GAPDH* mRNA, and the relative expression of each target gene was calculated with the formula n-fold change=2^−Δ*Ct*^, where Δ*C*_*t*_ represents *C*_*t*_ (infected)–*C*_*t*_ (control).^7^

### Mitochondrial Mass, mtDNA Content, and mtDNA Replication

Citrate synthase (CS) activity was measured as an indicator of mitochondrial mass. Briefly, sample homogenates were added to 100 mmol/L Tris-HCl (pH 8.0) and 6 mmol/L acetyl CoA, and CS catalyzed reduction of acetyl CoA (6 mmol/L) in the presence of oxaloacetate (10 mmol/L) in conjunction with 5,5′dithiobis-2-nitrobenzoic acid (10 mmol/L) reduction was monitored at 412 nm (ε13.6 m/mol/L per centimeter).^4^

Total DNA from cardiomyocytes (10^6^/sample) or tissue sections (5 mg) was isolated using a DNA extraction kit (Qiagen). We determined mtDNA content by real-time PCR using 20 ng of total DNA with primers amplifying the 16S and COI regions and normalized to 18S nuDNA. To assess the first step of mtDNA replication, that is, 7S RNA binding in the D-loop region and D-loop formation,^13^ we performed real-time PCR amplification of the 7S region using RNA extracts (free of DNA) and normalized to mtDNA amount. Next, we treated the DNA preparation with MnlI endonuclease (site: 5′-CCTC(N)7^−3′^), which selectively digests dsDNA, followed by real-time PCR for a segment in the CYTB and COI regions. The CYTB sequence is proximal to the D-loop and contains MnlI sites, whereas the COI region lacks the MnlI site. The ratio of the PCR products after the MnlI treatment for the CYTB region and COI indicated the amount of mtDNA committed to replication.^13^

### Western Blotting

Cell homogenates (15 μg) or cytosolic (15 μg) and nuclear (10 μg) protein fractions were resolved on denaturing 10% acrylamide gels and proteins transferred to PVDF membranes using a wet, vertical Criterion Blotter (Bio-Rad). Membranes were blocked with 5% nonfat dry milk (NFDM; Lab-Scientific) in 50 mmol/L Tris-HCl (pH 7.4), 150 mmol/L NaCl, and 0.05% Tween-20 (TBST) or 3% BSA (Santa Cruz) and then incubated overnight at 4°C with antibodies against CYTB (1:200), SDHA (1:1000), PGC-1α (1:1000), PPARγ (1:1000), ERRα (1:2500), ERRγ (1:50), NRF2 (1:200), TFB2M (1:400), 4-HNE (1:3000), SOD1 (1:200), or SOD2 (1:200). All antibody dilutions were made in 5% NFDM-TBST. After washing, membranes were incubated with the appropriate HRP-conjugated secondary antibody for 1 hour, and signal was developed using an enhanced chemiluminescence detection system (GE Healthcare). Membranes were stained with Coomassie blue G250 (Bio-Rad) to confirm an equal loading of samples. Images were visualized and digitized, and densitometry was performed using a FluorChem 8800 Image Analyzer System (Alpha Innotech).

### ROS Measurements

Supernatants (50 μL/well) from *T cruzi–*infected cardiomyocytes (0 to 24 hours) were added in triplicate to 96-well flat-bottomed plates and mixed with 50 μL of 100 μmol/L 10-acetyl-3,7-dihydroxyphenoxazine (Amplex red, Molecular Probes) and 50 μL of 0.3 U/mL horseradish peroxidase. The H_2_O_2_-dependent oxidation of Amplex red to red fluorescent resorufin (Ex_563nm_/Em_587nm_) was recorded using a SpectraMax M2 microplate reader (Molecular Devices).^6^

### Immunohistochemistry

Heart tissue sections were embedded in Tissue-Tek O.C.T and frozen. Cryostat sections (5 μm) were blocked with 5% normal goat or rabbit serum and then incubated at 4°C for 12 hours with antibodies against ND1, CYTB, CYPA, PGC-1α, PPARγ, NRF2, TFB2M, 4-HNE, 3-NT, 8-OHdG, SOD2, or HO-1 (all antibody dilutions 1:50). After washing, slides were incubated at room temperature for 1 hour each with biotinylated anti-goat or rabbit IgG (1:100 dilution) and streptavidin-conjugated alkaline phosphatase (1:100 dilution), and color was developed with a Red AP Kit I (Vector Labs). Tissue sections were counterstained with Mayer's hematoxylin to highlight the nuclei (blue). Each tissue section was analyzed for at least 5 microscopic fields on an Olympus polarizing microscope (Center Valley, PA) and scored for staining as a percentage of total histological field quantified using MetaMorph^R^ Microscopy Automation & Image Analysis Software (Molecular Devices).

### Fluorescence Microscopy

Cardiomyocytes were cultured in Nunc^R^ Lab Tek II chamber slides and incubated with *T cruzi* for 0 to 24 hours. Cells were washed, fixed with ice-cold acetone/methanol (1:1 v/v), blocked with 1% BSA, and then incubated overnight at 4°C with mouse anti-8OHdG (IgM) and mouse anti-SDHA (IgG) antibodies (1:50 dilution). Slides were washed, incubated with phycoerythrin-conjugated goat anti-mouse IgM (1:100, Santa Cruz) and FITC-conjugated goat anti-mouse IgG (1:100, Sigma) for 1 hour in the dark, and counterstained with DAPI (binds DNA). Fluorescence was monitored on an Olympus BX-15 fluorescence microscope equipped with a digital camera (magnification ×40).

### Statistical Analysis

In vitro experiments were conducted 3 times with triplicate observations/sample/time. In vivo experiments were conducted twice with duplicate observations per sample (n=6/group). Data are presented as mean±SD. Normally distributed data were analyzed by the Student *t* test (comparison of 2 groups) and 1-way ANOVA with Tukey's post hoc test (comparison of multiple groups). The nonparametric Mann–Whitney *U* test (also called the Wilcoxon rank-sum test) and the Kruskal–Wallis test were used to analyze statistical significance for data that were not normally distributed. Significance for all tests was accepted at *P*<0.05.

## Results

**Figure 1.**
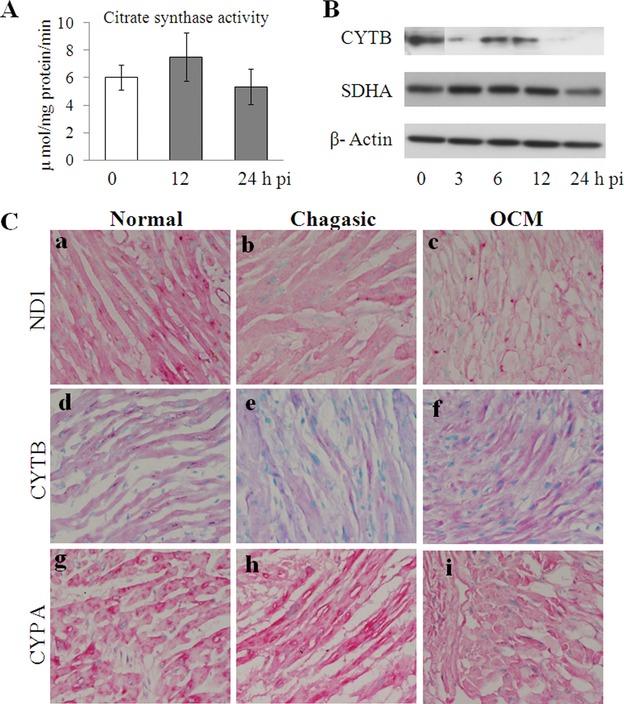
Mitochondrial protein content, but not mass, is decreased in infected cardiomyocytes and chagasic hearts. A and B, Cardiomyocytes were infected with *Trypanosoma cruzi* for 0 to 24 hours: (A) citrate synthase activity; (B) Western blotting for CYTB, SDHA, and β-actin; (C) cryostat sections of the heart biopsies from normal healthy donors (a, d, g), chagasic patients (b, e, h), and other cardiomyopathy (OCM) patients (c, f, i) were submitted to immunostaining for ND1 (a-c), CYTB (d-f), and CYPA (g-i) (magnification ×20). Data in all bar graphs are presented as mean±SD. Statistical significance of normally distributed data was analyzed by the Student *t* test/ANOVA/Tukey's test; *P<0.05, **P<0.01, ***P<0.001. In some bar graphs, statistical significance by the nonparametric Kruskal–Wallis test is also presented (horizontal dotted line). SD indicates standard deviation; ANOVA, analysis of variance.

Experimental animals infected by *T cruzi* exhibited decreased respiratory chain activity (reviewed in reference ^14^). To assess if mitochondrial dysfunction occurs in chagasic patients, we examined mitochondrial markers at the levels of enzyme activity and gene and protein expression in infected cardiomyocytes and cardiac biopsies. Citrate synthase activity (a mitochondrial matrix protein) was not significantly changed in infected cardiomyocytes (Figure 1A). The cellular protein level of mtDNA-encoded CYTB (complex III subunit) and nuDNA-encoded SDHA (complex II subunit) was decreased by 65% (*P*<0.01_ANOVA-Tukey's_) and 20%, respectively, in cardiomyocytes 24 hours postinfection (pi; Figure 1B).

Immunohistochemistry of cardiac biopsies exhibited mtDNA-encoded ND1 (complex I subunit) decreased by 70% in chagasic and other cardiomyopathy (OCM) patients, and CYTB was decreased by 40% in chagasic patients (Figure 1C, Table 2, all *P* values <0.05_Mann-Whitney_). No change was noted in tissue staining for nuDNA-encoded CYPA. These results suggest that mitochondrial mass was not changed, but mitochondria-encoded proteins were significantly decreased in human cardiomyocytes (by 24 hours pi) and chronically infected chagasic hearts.

**Table 2.** Semiquantitative Scoring of Immunostaining of Cardiac Tissue Biopsies

	Arbitrary Scores, Mean (Range)		
	
Immunostaining	Normal	Chagasic	Other Cardiomyopathy
Figure 1			
			
CYTB	12.5 (9.3 to 16.8)	7.4 (1.7 to 16.4)*^††^	22.0 (1.7 to 40.0)*

ND1	54.6 (2.8 to 26.7)	16.4 (1.5 to 56.5)*^‡^	1.9 (0.2 to 4.6)*^§§§^

CYPA	141 (45.0 to 452)	177 (24.1 to 380)	104 (34.2 to 182)

Figure 3			
			
PGC-1α	1399 (1141 to 1963)	273.9 (5.7 to 1458.8)**^‡§§§^	792 (156 to 2557)**^§^

PPARγ	60.1 (3.6 to 369)	6.0 (0.2 to 20.9)***^§§§^	1.4 (0.5 to 2.2)*^§§^

NRF2	1017 (505 to 1798)	846(112 to 1461)*	766 (690 to 895)

Figure 6			
			
4-HNE	12.8 (1.3 to 54.3)	197.4 (53.4 to 817)***^‡§§§^	632 (116 to 1934)**^§§§^

3-NT	7.3 (2.1 to 14.8)	416.0 (173.6 to 711.6)***^‡§§§^	260 (4.0 to 1027)**^§^

8-OHdG	376 (76 to 883)	725 (437 to 1442)*	1099.3 (687 to 1442)^§^

Figure 7			
			
SOD2	1228(119 to 4629)	763 (156 to 3599)*	1035 (212 to 2651)

HO-1	431(39.4 to 2529)	4.3 (1.3 to 9.4)*^‡§§§^	111 (5.0 to 527)**^§^

Cardiac biopsies of normal donors and of chagasic and other cardiomyopathy patients (n=6 to 8/group) were fixed in OCT, and 5-μm-thick cryostat sections (5 sections/tissue) were submitted for immunostaining, as described in Materials and Methods. Each slide was analyzed for 5 microscopic fields on an Olympus polarizing microscope (Center Valley, PA), and staining for primary antibody was scored as a percentage of total histological fields quantified using MetaMorph Microscopy Automation & Image Analysis Software (Molecular Devices). The Mann–Whitney test was employed for pairwise comparison.

Significance by t tests is presented as *normal versus disease (chagasic or other cardiomyopathy); ^‡^chagasic versus other cardiomyopathy.

Significance by the Kruskal–Wallis Dunn's test is presented as ^§^normal versus chagasic; ^§^normal versus other cardiomyopathy; ^†^chagasic versus other cardiomyopathy groups.

Significant at *^‡§^*P* < 0.05, **^††§§^*P* < 0.01, ***^§§§^*P* < 0.001.

**Figure 2.**
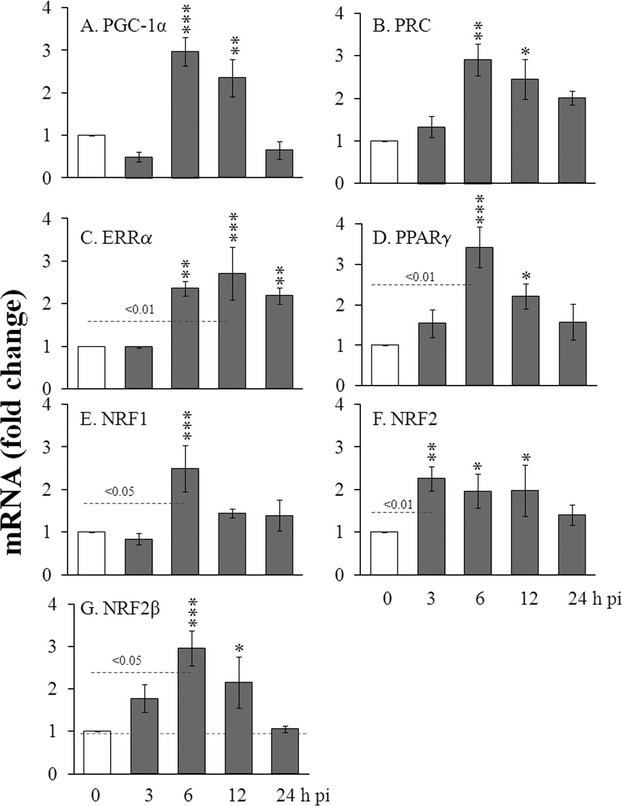
Gene expression of PGC-1 transcriptional cascade in *T cruzi–*infected cardiomyocytes. Cardiomyocytes were infected with *Tryapnosoma cruzi* (1:3 cell:parasite ratio) for 0 to 24 hours. Shown are the relative changes in gene expression for PGC-1, PRC, and the coactivated transcription factors (ERRα, PPARγ, NRF1, NRF2, and NRF2β), determined by real-time RT-PCR. Results were normalized to GAPDH mRNA and represent mean values obtained from at least 2 independent experiments (triplicate observations/experiment). Statistical significance of data was analyzed as described in Figure 1.PGC indicates peroxisome proliferator activated receptor gamma coactivator-1; ERR, estrogen-related receptor; PPAR, peroxisome proliferator-activated receptor; NRF, nuclear respiratory factor; RT-PCR, reverse-transcription polymerase chain reaction.

PGC-1α serves as a regulator of mitochondrial biogenesis and thereby plays a key role in maintaining mitochondrial functional capacity.^15^ We determined expression of the PGC-1 family members and coactivated transcriptional factors involved in mitochondrial biogenesis to assess whether the PGC-1 transcriptional cascade is compromised during *T cruzi* infection (Figure 2). The mRNA levels for PRC and ERRα were increased by 2- to 3-fold in cardiomyocytes during 6 to 24 hours pi (Figure 2B and 2C, *P*<0.05_ANOVA-Tukey's_). The mRNA levels for PGC-1α, PPARγ, NRF1, and NRF2β were increased by 2.5- to 3.5-fold 6 hours pi (all *P*<0.001_ANOVA-Tukey's,_ *P*<0.05_Kruskal–Wallis_), after which a downward trend, reaching basal level expression by 24 hours pi, was noted. The increase in mRNA for NRF2 in infected cardiomyocytes was stable during 3 to 12 hours (*P*<0.001_ANOVA-Tukey's_, *P*<0.05_Kruskal–Wallis_) and normalized 24 hours pi (Figure 2F). These data suggested that cardiomyocytes responded to *T cruzi* infection by (1) increased mRNA levels for PGC-1α, PRC, and coactivators (PPARγ, ERRα, NRF1/NRF2), and (2) PGC-1α and NRF1/2 upregulation was abolished 24 hours pi when maximal decline in mtDNA-encoded proteins was observed.

**Figure 3.**
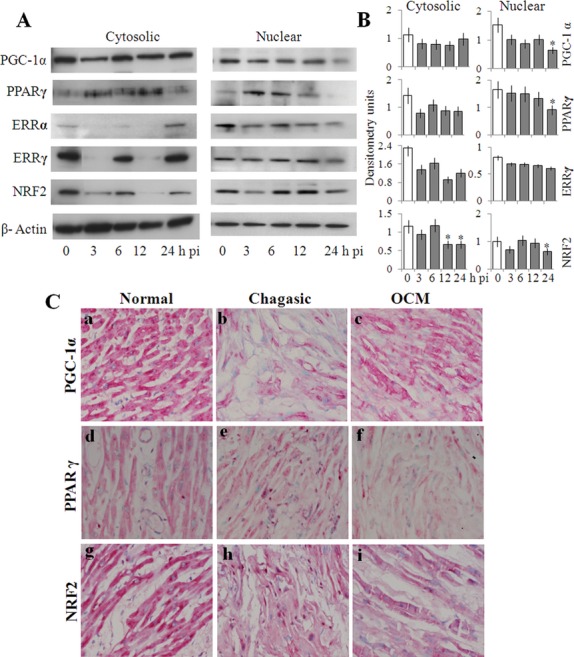
Protein level and nuclear translocation of PGC-1-activated transcription factors in infected cardiomyocytes and chagasic hearts. A and B, Western blots for cytosolic and nuclear protein levels for PGC-1α and coactivated transcriptional factors in cardiomyocytes, harvested 0 to 24 hours postinfection (A) was quantified by densitometry (B). C, Representative immunostaining images for PGC-1α (a-c), PPARγ (d-f), and NRF2 (g-i) in cardiac biopsies of normal donors (a, d, g), and chagasic (b, e, h) and OCM patients (c, f, i). Statistical significance of data was analyzed as described in Figure 1. PGC indicates peroxisome proliferator-activated receptor gamma coactivator-1; ERR, estrogen-related receptor; PPAR, peroxisome proliferator-activated receptor; NRF, nuclear respiratory factor; OCM, other cardiomyopathy.

We performed Western blotting for PGC-1α and downstream transcriptional factors involved in mitochondrial biogenesis to determine if mRNA levels correlated with protein levels and if nuclear transport of 1 or more of these factors was compromised in infected human cardiomyocytes and heart. The cytosolic level of PGC-1α in infected cardiomyocytes was almost stable, whereas PGC-1-activated transcription factors exhibited up- or downregulation during the 3- to 24-hour pi period (Figure 3A and 3B). In comparison, nuclear levels of PGC-1α and coactivated transcription factors (PPARγ, ERRα, ERRγ, and NRF2) exhibited a downward trend during the 6 to 24 hours pi, with maximal decline (30% to 60%) noted 24 hour pi (Figure 3A and 3B, *P*<0.05_ANOVA-Tukey's_). Immunostaining showed a majority of the PGC-1α, PPARγ, and NRF2 were localized to nuclei in chagasic heart biopsies (Figure 3C). The overall expression for PGC-1α was markedly decreased in chagasic hearts, whereas PPARγ and NRF2 were similarly decreased in chagasic and OCM patients (Figure 3C, Table 2) and corroborated the in vitro observations in infected cardiomyocytes.

**Figure 4.**
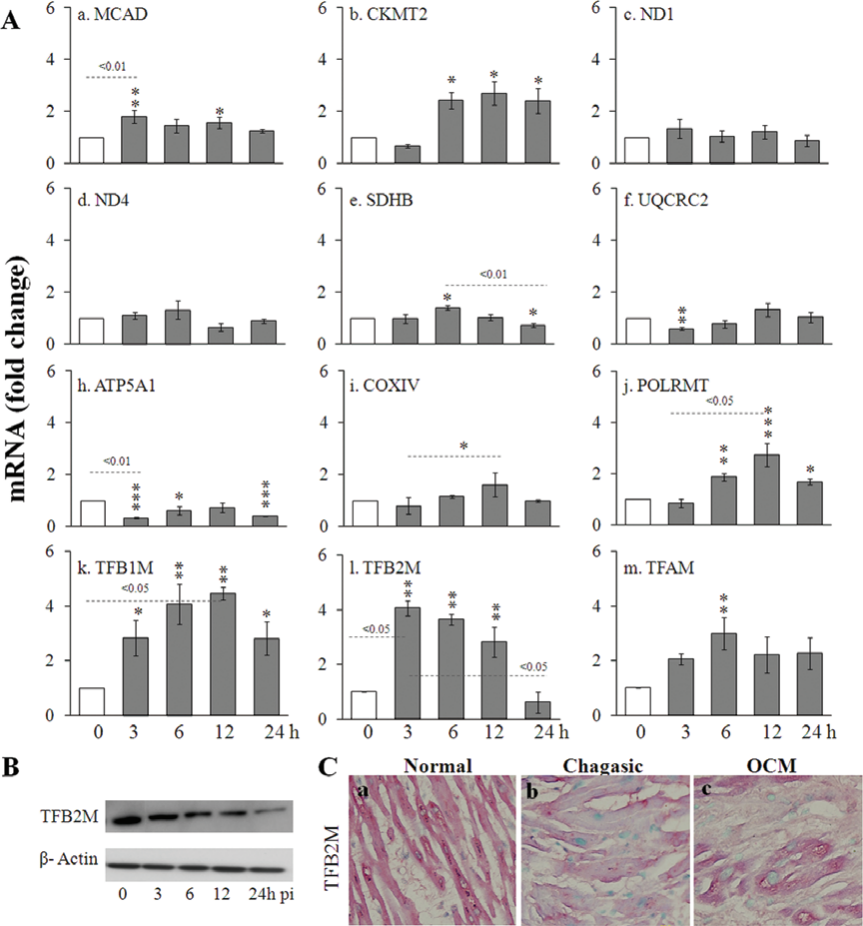
Expression of genes related to mtDNA-encoded transcripts and mitochondrial transcriptional machinery in *T cruzi–*infected cardiomyocytes. A, Cardiomyocytes were harvested 0 to 24 hours postinfection. mRNA levels for PGC-1α downstream gene targets mediated by ERRs/PPARγ (a, b) and ERR/NRFs (c-m) that are involved in fatty acid oxidation (a), energy transport (b), OXPHOS (c-i), and mitochondrial transcription (f-m) were monitored by real-time RT-PCR. Data are shown as the mean of the fold changes over normal controls±SD and normalized by GAPDH mRNA. B, Western blotting for TFB2M in infected cardiomyocytes. C, Representative immunostaining images for TFB2M in heart tissue biopsies. Statistical significance of data was analyzed as described in Figure 1. PGC indicates peroxisome proliferator-activated receptor gamma coactivator-1; ERR, estrogen-related receptor; PPAR, peroxisome proliferator-activated receptor; NRF, nuclear respiratory factor; OCM, other cardiomyopathy; SD, standard deviation; RT-PCR, reverse-transcription polymerase chain reaction.

We assessed the functional significance of a decline in expression and/or nuclear transport of PGC-1α and its coactivated targets by evaluating the expression of their downstream genes involved in mitochondrial function and biogenesis. The expression of genes under PPAR control, for example, *MCAD*, involved in fatty acid metabolism, and *CKMT2*, which transports energy from mitochondria to the cytosol, was increased by 25% to 80% and 2.5- to 3-fold, respectively, in infected cells (*P*<0.05_ANOVA-Tukey's_; Figure 4A.a,b). Gene expression for *ND1, ND4, UQCRC2, COXIV,* and *ATP5A1*, the components of respiratory complexes essential for maintaining OXPHOS and suggested to be controlled by NRF2, was mostly nonresponsive 3 to 12 hours pi in infected cardiomyocytes, and remained below or at par level with that noted in normal controls (Figure 4A.c-i). The nonresponsiveness of OXPHOS-related transcripts could be a result of changes in mitochondrial transcription efficiency, also controlled by PGC-1α-activated NRF1/2. Our data showed that mRNA levels of POLRMT, TFB1M, TFB2M, and TFAM of the mitochondrial transcription machinery were increased by 2.7-fold, 4.5-fold, 4-fold, and 3-fold, respectively, during 3 to 12 hours pi (all *P*<0.05_ANOVA-Tukey's_; Figure 4A.j-m). Only TFB2M exhibited a significant decline, both at the mRNA (Figure 4A.l) and protein (Figure 4B) levels, in *T cruzi*–infected cardiomyocytes 24 hours pi (*P*<0.05_Kruskal–Wallis_). Chagasic heart biopsies also exhibited a significant decline in TFB2M immunostaining (Figure 4C). These results suggested that coactivation function of PGC-1α and NRF2-driven expression of genes involved in mitochondrial gene transcription were maintained at least early during the course of infection and likely were not responsible for the nonresponsiveness of OXPHOS-related gene expression in cardiomyocytes and human heart infected by *T cruzi*.

**Figure 5.**
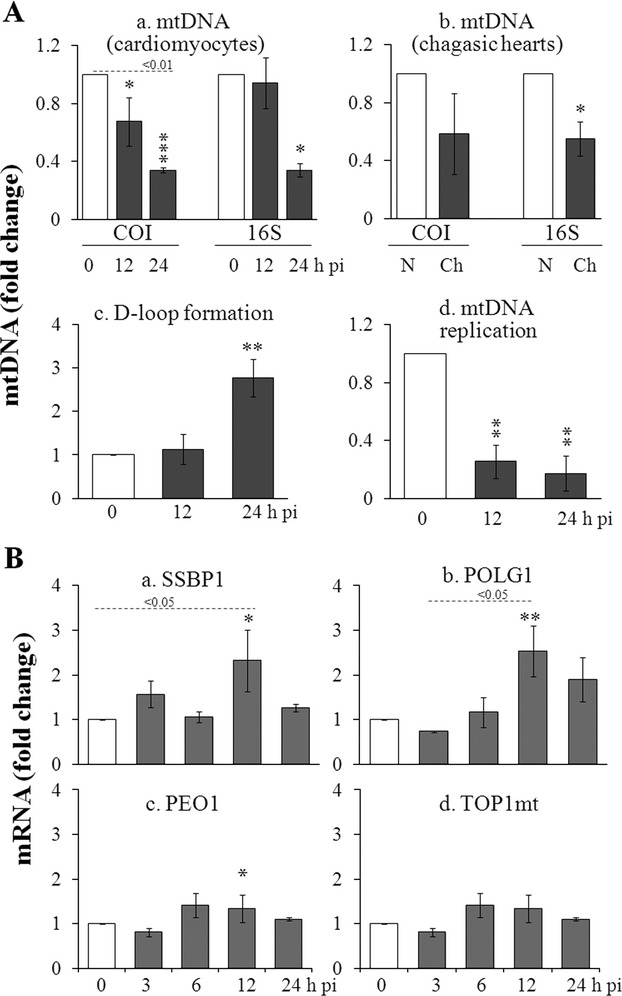
Defects of mtDNA replication cause mtDNA deficiency during *T cruzi* infection. A, mtDNA content in *T cruzi–*infected human cardiomyocytes (a) and cardiac biopsies of chagasic patients (b) was determined by real-time PCR amplification of COI and 16S regions of mtDNA, normalized to 18S nuDNA. D-loop formation was assessed by measuring 7S RNA, normalized to mtDNA amount in infected cardiomyocytes (c). mtDNA replication was assessed by measuring the extension of 7S DNA beyond the D-loop (d). B, Real-time RT-PCR amplification of mRNA for PGC-1/NRF gene targets (*SSBP1, POLG, PEO1*, and *TOP1mt*) involved in mtDNA replication in *T cruzi–*infected cardiomyocytes. Statistical significance of data was analyzed as described in Figure 1. RT-PCR indicates reverse-transcription polymerase chain reaction; SSBP, single-stranded DNA-binding protein.

Next, we examined whether mtDNA availability for carrying out transcription of mtDNA-encoded genes was compromised in infected cardiomyocytes. The mtDNA level, determined by COI and16S contents normalized to 18S nuDNA, was decreased by 65% in infected cardiomyocytes 24 hours pi (*P*<0.001_ANOVA-Tukey's_ and *P*<0.05_Kruskal–Wallis_; Figure 5A.a) and by 40% in chagasic heart biopsies (*P*<0.05_ANOVA-Tukey's_; Figure 5A.b). To substantiate the finding of decreased mtDNA and to identify the mechanisms for reduced mtDNA levels, we investigated mtDNA replication capacity in infected cardiomyocytes. The first step in mtDNA replication is the formation of the D-loop at the origin of replication. The synthesis of 7S RNA (normalized to mtDNA content) suggests D-loop formation and was increased 2.8-fold in infected cardiomyocytes (*P*<0.001_ANOVA-Tukey's_; Figure 5A.c) compared with normal controls. However, extension of 7S DNA furthering mtDNA replication was substantially reduced, evidenced by >75% decline in single-stranded CYTB versus mtDNA (COI) in infected cardiomyocytes (*P*<0.001_ANOVA-Tukey's_; Figure 5A.d). The increase in D-loop formation in infected cardiomyocytes was consistent with preserved or enhanced mRNA for single-stranded DNA-binding protein (SSBP1) involved in the initiation of replication (Figure 5B.a). The decline in mtDNA replication occurred despite the observation that the mRNA levels of genes forming the DNA replication complex, that is, *POLG1, PEO1*, and *TOP1mt*, were normal or higher in infected cardiomyocytes than in normal controls (Figure 5B.b-d). These results suggest that mtDNA replication machinery and initiation of mtDNA replication were intact; however, extension of mtDNA strands was defective and resulted in decreased mtDNA content in infected cardiomyocytes and chagasic hearts.

**Figure 6.**
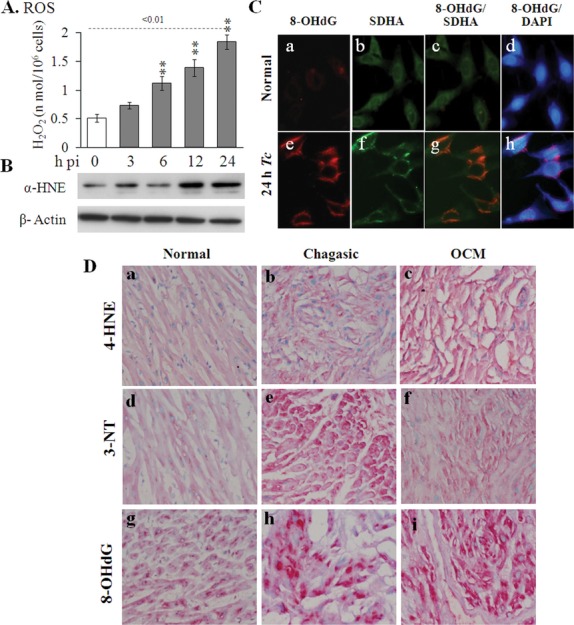
Oxidative stress-induced mtDNA damage was increased in infected cardiomyocytes and chagasic hearts. A-C, Cardiomyocytes were infected with *Trypanosoma cruzi* for 0 to 24 hours. A, ROS release was determined by a Amplex red assay. B, Cell lysates were subjected to Western blotting with anti-4-hydroxynonenal antibody (control: anti-β-actin antibody). C, Immunofluorescence for 8-OHdG (a, e) and SDHA (b, f) in normal and infected cells (24 hours postinfection). Overlay (c, g) shows mitochondrial localization of 8-OHdG, also confirmed by counterstaining of anti-8OHG-stained cells with DAPI (d, h). D, Immunostaining for 4-HNE (a-c), 3-nitrotyrosine (d-f), and 8-OHdG (g-i) in heart biopsies of normal donors (a, d, g) and chagasic (b, e, h) and OCM (c, f, i) patients. Statistical significance of data was analyzed as described in Figure 1. ROS indicates reactive oxygen species; OCM, other cardiomyopahy.

We considered that enhanced oxidative stress, noted in chagasic mice, might be a possible cause of impaired DNA replication in infected human cardiomyocytes and heart. The levels of reactive oxygen species (ROS; *P*<0.01_ANOVA-Tukey's_; Figure 6A) and 4-hydroxynonenal (4-HNE; *P*<0.05_ANOVA-Tukey's_; Figure 6B), which is an oxidative stress marker, were progressively increased in infected cardiomyocytes during 6 to 24 hours post infection. 8-Hydroxydeoxyguanosine (8-OHdG) is an oxidized nucleoside of DNA and the most frequently detected DNA lesion. Overlay of immunofluorescence for 8-OHdG and SDHA (mitochondrial matrix protein) showed 8-OHdG was primarily localized in mitochondria surrounding the nucleus in infected cardiomyocytes (Figure 6C.g), also verified by counterstaining of 8-OHdG-labeled infected cells with DAPI (accumulates in the nucleus; Figure 6C.h). Similar to the observations made in *T cruzi–*infected cardiomyocytes, chagasic heart biopsies exhibited a substantial increase in 4-HNE and 3-nitrotyrosine markers of oxidative and nitrosative stress and up to a 2-fold increase in 8-OHdG levels (Figure 6D). A comparable increase in oxidative stress was noted in cardiac biopsies of OCM patients. Overall, the results presented in Figure 6 suggest that oxidative stress and DNA damage were increased during chagasic and other cardiomyopathies, and mtDNA (not nuDNA) is primarily susceptible to oxidative stress–induced damage in *T cruzi–*infected cardiomyocytes.

**Figure 7.**
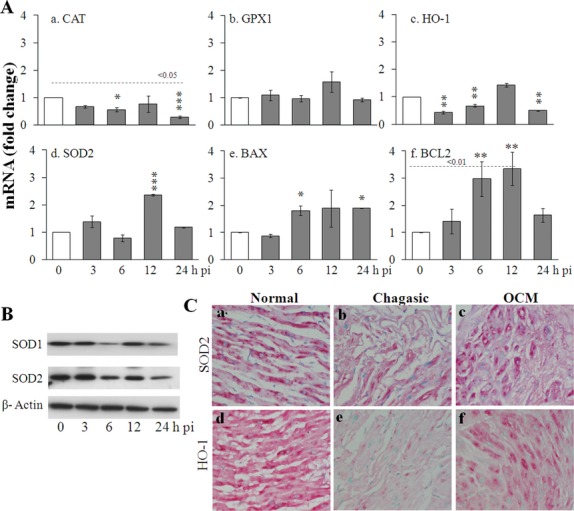
Antioxidant gene expression is nonresponsive or compromised in infected cardiomyocytes and chagasic hearts. A, Real-time RT-PCR determination of PGC-1α/NRF2 gene targets for antioxidant response (a-d) and apoptosis (e, f) in *T cruzi–*infected cardiomyocytes 0 to 24 hours postinfection. B, Western blotting for SOD1 and SOD2 antioxidant isoforms in infected cardiomyocytes. C, Immunohistochemistry for SOD2 and HO-1 in heart biopsies of normal donors and chagasic and OCM patients. Statistical significance of data was analyzed as described in Figure 1. PGC indicates peroxisome proliferator-activated receptor gamma coactivator-1; OCM, other cardiomyopahy; RT-PCR, reverse-transcription polymerase chain reaction; NRF, nuclear respiratory factor.

PGC-1α-activated NRF1/2 transcription factors regulate antioxidant and cell survival gene expression. Despite a substantial increase in ROS levels and oxidative stress, NRF2-regulated transcription of genes encoding catalase, GPX1, and HO-1 antioxidants were suppressed or unaltered, and mRNA for *SOD2* (mitochondrial) was marginally increased 12 hours pi (Figure 7A.a-d). The mRNA ratio for *BCL2/BAX* was decreased by 24 hours pi (Figure 7A.e,f), indicating cells were committed to apoptotic death. At the protein level, we observed no change or a decline in SOD1 and SOD2 in infected cardiomyocytes during 3 to 24 hours pi (Figure 7B). Immunostaining for SOD2 and HO-1 was decreased by 40% to 90% in cardiac biopsies of chagasic (Figure 7C.b,e, Table 2) but not in OCM (Figure 7C.c,f) patients. The results in Figure 7 suggest that the antioxidant/cell survival response was compromised in *T cruzi–*infected cardiomyocytes and chagasic hearts.

## Discussion

We have performed *in vitro* and *in vivo* studies to investigate the mechanisms of mitochondrial dysfunction in human Chagas disease. We have found that mtDNA replication was significantly compromised and caused deficiency of mtDNA content and expression of OXPHOS genes in *T cruzi–*infected human cardiomyocytes and chagasic hearts. The decline in mtDNA replication did not appear to be a result of defects in the expression of PGC-1α, PGC-1-coactivated transcription factors, downstream target genes of mitochondrial transcription machinery that provide single-stranded RNA to initiate mtDNA replication, or the genes of replication machinery that carry out mtDNA replication. Instead, we found that the defects in mtDNA replication were a result of increased ROS generation and oxidative stress that caused mtDNA oxidation, rendering it unfit for maintaining replication and expression of OXPHOS genes. We also found that the selective functional incapacity of NRF2-mediated antioxidant gene expression contributed to increased oxidative stress in infected cardiomyocytes, providing an atmosphere for oxidative stress–induced mtDNA damage. To the best of our knowledge, this is the first study demonstrating a molecular mechanism for mitochondrial functional decline in human Chagas disease.

The heart is highly dependent on mitochondria for the energy required for its contractile and other metabolic activities. Mitochondria represent 30% of the total volume of cardiomyocytes and provide ≍90% of the cellular ATP energy through the OXPHOS pathway. In experimental mice and rats infected by *T cruzi*, the expression of mitochondrial function-related transcripts^16,17^ and, consequently, the activities of respiratory complexes, NADH-ubiquinone reductase (CI), ubiquinol-cytochrome c reductase (CIII),^4^ and ATP synthase (CV)^18^ were decreased in the myocardium. The functional effect of these perturbations was evidenced by decreased mitochondrial respiration^7,19^ and reduced myocardial and mitochondrial ATP levels,^5^ and these defects were sustained with progressive development of cardiomyopathy in infected animals.^5,20^ Our observations of an overall decline in OXPHOS-related gene expression at the mRNA and protein levels in *T cruzi–*infected human cardiomyocytes and cardiac biopsies of chagasic patients (Figures 1 and 4) provide the first molecular evidence of mitochondrial functional decline in human Chagas disease and support the previously reported observation of a decline in respiratory complex activities in the peripheral blood of chagasic patients.^21^

Stress-responsive PGC-1α is a member of the PGC-1 family and suggested to play a role in mitochondrial biogenesis, fatty acid metabolism, and OXPHOS via activation of specific transcription factors.^10,12^ For example, PPAR family members (α, β, γ) are key regulators of genes involved in lipid metabolism and are expressed in tissues with high rates of mitochondrial fatty acid oxidation, such as heart and skeletal muscle.^22,23^ NRF1/2, the nuclear-encoded respiratory factors, are shown to drive transcription of genes involved in OXPHOS and transcription and replication of the mitochondrial genome.^12^ PGC-1 coactivates both these transcription factors as well as the ERR (α, β, δ) family of transcription factors. The current models suggest that ERRs cooperate with or directly activate NRF1/2 and PPARs serving as an amplifier for PGC-1α coactivation of the OXPHOS pathway and fatty acid oxidation while inhibiting glucose oxidation.^24^ In mouse models, PGC-1α expression is directly correlated to metabolic functional state; mice with inducible cardiac-specific overexpression of PGC-1α showed a robust mitochondrial biogenic response on activation by stress stimuli,^25^ and PGC-1α-knockout mice developed cardiomyopathy.^26^ PGC-1α downregulation was associated with skeletal muscle catabolic wasting,^27^ and endurance exercise training, known to promote oxidative phenotype, stimulated PGC-1α expression in skeletal muscle of humans.^28^ These studies suggest that PGC-1-coactivated transcriptional factors provide the key mechanism for master regulation of mitochondrial biogenesis and function and may also be dysregulated in chagasic conditions. Our findings in this study showed that the mRNA and cytosolic protein levels of PGC-1α, PPARs, ERRs, and NRF1/2 in *T cruzi–*infected cardiomyocytes were initially increased and then returned to basal level or were decreased by 24 hours pi (Figures 2 and 3), indicating that chronic infection would dysregulate the expression of PGC-1 and PGC-1-coactivated transcription factors. This was supported by the observed decline in PGC-1α, PPARγ, and NRF2 in heart biopsies of chronically infected chagasic patients (Figure 3). Despite this, the nuclear localization of PGC-1α, ERRs, PPARγ, and NRF2 proteins in the heart biopsies of chagasic patients was increased compared with that in normal controls (Figure 3), suggesting that a physiological deficiency of transcription of downstream target genes would not ensue in chagasic hearts. Indeed, PPARγ/ERRα-regulated expression of genes of fatty acid metabolism (eg, *MCAD*) and energy transport (eg, *CKMT2*) was enhanced >2-fold in infected cardiomyocytes (Figure 4). Likewise, NRF1/2-regulated expression of genes involved in transcription (eg, *TFB1M, TFB2M, TFAM*) and replication (eg, *SSBP1, POLG1*) of the mitochondrial genome was enhanced in infected cardiomyocytes or similar to that detected in normal controls (Figure 4). These observations suggested that PGC-1α coactivation function is, in general, not compromised and likely not the key to mitochondrial impaired function in chagasic cardiomyopathy. Others have also reported that PGC-1α downregulation does not uniformly present in human heart failure of other etiologies.^29^ Thus, our studies indicate that further activation of PGC-1α may not be beneficial in chagasic cardiomyopathy and may actually cause harm, as is noted in mice in whom overexpression of PGC-α resulted in tremendous mitochondrial biogenesis, leading to cardiomyopathy.^25,30^

NRF2, in addition to regulating the expression of genes involved in mitochondrial transcription and replication, is known to modulate antioxidant gene expression.^31^ We noted the NRF2-regulated expression of antioxidants (eg, CAT, GPX1, HO-1) was nonresponsive to increased oxidative stress in infected cardiomyocytes and chagasic hearts, also supported by experimental studies in mice demonstrating the nonresponsiveness of glutathione antioxidant defense during chronic Chagas disease.^8^ How *T cruzi* may modulate NRF2 is not known; however, we can speculate that parasite expression of antioxidants (eg, tryparedoxin peroxidases)^32^ may provide autocrine regulation of NRF2, setting the stage for oxidative stress in the host. Indeed, pro-oxidant milieu of chagasic hearts in experimental mice is evidenced by increased levels of ROS, GSSG, and lipid and protein oxidation.^5,8^ A pro-oxidant status in human chagasic patients is documented on the basis of a systemic increase in peripheral GSSG and MDA content,^33,34^ further supported by our observations of increased oxidative stress (4-HNE, 3-NT) in infected cardiomyocytes and human heart (Figure 6). Moreover, the treatment of *T cruzi*–infected animals with an antioxidant tipped the balance in favor of preserving mitochondrial and cardiac function,^5,20^ thus supporting the idea that antioxidant depletion or inefficient scavenging of ROS is linked to mitochondrial dysfunction in Chagas disease. In this study, we have provided the first observation that mtDNA is oxidized (Figure 6), and an increase in mtDNA oxidation is of pathological importance in human Chagas disease. This is because oxidized mtDNA was not fit for completing replication. Our data showed that mtDNA replication, that is, D-loop formation, was initiated at an almost 2-fold higher rate than that noted in controls; however, the infected host exhibited significant disability in completing mtDNA replication (Figure 5), leading to decreased capacity to maintain mtDNA content and expression of mtDNA-encoded genes of the OXPHOS pathway (Figure 4). Our observations are consistent with some studies suggesting that NRF1/2 activation prevents diabetic cardiomyopathy,^35^ whereas they refute others who have reported ERR downregulation as a key mechanism in decreased expression of PGC-1 target genes in heart failure.^29^ We surmise that future studies identifying the mechanism of selective dysregulation of NRF2-dependent antioxidant gene expression will provide clues to therapies for the restoration of mitochondrial function in chagasic hearts.

In summary, we have shown that infected cardiomyocytes and chagasic hearts are severely impaired in mitochondrial biogenesis, evidenced by decreased mtDNA content. We have identified the potential mechanisms for mtDNA depletion in the heart. First, our data suggest that mtDNA replication was severely impaired, resulting in a significant loss of mtDNA and mtDNA-encoded proteins of OXPHOS pathway. Second, we found that mtDNA replication defects were associated with increased ROS generation and selective functional incapacity of NRF2-mediated antioxidant gene expression. Our data suggest that oxidation of mtDNA rendered it unfit for replication and gene expression. The PGC-1α-coactivated NRF1/2 transcriptional activity in the expression of genes of mitochondrial transcription and replication machinery was not compromised. Overall, our studies provide a basis for investigating the novel mechanisms of chagasic heart disease and designing therapies targeting restoration of NRF1/2 function in maintaining antioxidant status to prevent heart failure.
